# The ethical relevance of the unconscious

**DOI:** 10.1186/s13010-017-0053-9

**Published:** 2017-12-29

**Authors:** Michele Farisco, Kathinka Evers

**Affiliations:** 1 Centre for Research Ethics and Bioethics, Uppsala University, Uppsala, Sweden; 2 Brain Project Sub-Project 12, Uppsala University, Uppsala, Sweden; 3 Biogem, Genetic Research Centre, Ariano Irpino, (AV) Italy

**Keywords:** Brain, Consciousness, Disorders of consciousness, The unconscious, Ethics, Neuroethics

## Abstract

**Background:**

Ethical analyses of disorders of consciousness traditionally focus on residual awareness. Going one step further, this paper explores the potential ethical relevance of the unawareness retained by patients with disorders of consciousness, focusing specifically on the ethical implications of the description of the unconscious provided by recent scientific research.

**Methods:**

A conceptual methodology is used, based on the review and analysis of relevant scientific literature on the unconscious and the logical argumentation in favour of the ethical conclusions.

**Results:**

Two conditions (experiential wellbeing and having interests) that are generally considered critical components in the ethical discussion of patients with disorders of consciousness might arguably be both conscious and unconscious.

**Conclusions:**

The unconscious, as well as consciousness, should be taken into account in the ethical discussions of patients with disorders of consciousness.

## Introduction

Contemporary cognitive science increasingly reveals that the traditional way of depicting the unconscious as a dimension deeply separated from and even opposed to consciousness is misleading and overly simplistic [1]. Unawareness^1^ is far more than a passive repository of information: a lot of monitory work (i.e., active exploration of the environment) takes place at the unaware level, which shows remarkable similarities with awareness as well as a deep connection with it. From a neurophysiological point of view, awareness (consciousness) is what psychoanalysis claimed a century earlier: merely “the tip of the mental iceberg” [2](p. 17).

We explore the ethical relevance of the unconscious and its possible clinical implications for the assessment and care of patients with disorders of consciousness (DOCs), e.g. coma, vegetative state/unresponsive wakefulness syndrome, minimally conscious state [3–5]. Our aim is to show that not only awareness but also unawareness should be taken into account in the ethical analyses of DOCs. Two arguments are provided to support this view: one strong and one weak. The strong argument says that unawareness is ethically relevant because it has (at least in part) those characteristics of awareness that are usually considered critical in contemporary ethical debates about DOCs. In other words, if consciousness is ethically relevant by virtue of certain characteristics, and the unconscious shares those characteristics, then the unconscious is *ipso facto* ethically relevant.

The weak argument says that if consciousness is considered ethically relevant for the assessment of a patient with DOCs, and the unconscious is (at least in part) the result of consciousness and also heavily affects the development of consciousness (i.e., there is a relation of mutual influence between the two [6] (p. 112–114, cf. further below), then the unconscious is also ethically relevant.

The term ‘ethical relevance’ is here used in the same sense that Goodpaster uses the term ‘moral considerability’ (i.e., deserving moral consideration) as distinguished from moral significance (i.e., valuable, characterized by a specific moral value) [7]. Qualifying the unconscious as ethically relevant does accordingly not imply that it is also ethically valuable.

Both the abovementioned arguments stress the need to include unawareness in the ethical analysis of patients with DOCs. We suggest that the actual default way of setting ethical issues arising from DOCs (i.e., checking whether patients retains some aware abilities) is limited, and that this limitation is ethically and clinically questionable.

## Background

### Recent descriptions of the unconscious

Recent neuroscientific evidence shows that our brain is able to do a lot of things without requiring any actual aware experience by the subject. For instance, the brain is able to, without awareness, correlate information, associate meanings, reason in a very fast way, develop complex computations, perform sophisticated mathematical operations, selectively focus on information, develop complex inferences [8], and even perceive the affective value of stimuli and to influence motivation, value judgment and goal-directed behaviour [9].

Examples of activities traditionally associated with aware consciousness but showed to be feasible also at unaware levels are reading and doing arithmetic [10]. Using a technique called Continuous Flash Suppression (CFS), which consists of the presentation of a target stimulus to one eye and the simultaneous presentation of rapidly changing masks to the other eye, allowing subliminal presentations that last seconds, Sklar and colleagues have shown that even quite sophisticated human cultural products, such as semantically processing a number of words and solving arithmetic equations, can take place outside explicit awareness. Participants were presented with semantically coherent and semantically non-coherent expressions. The latter appeared to awareness before the former, showing the ability of the brain to semantically process them outside explicit awareness.

The amount of operations that the brain is able to perform at the unaware level is impressive. Summarizing empirical evidence, Hassin is quite radical in concluding that the unconscious is able to perform every fundamental high-level cognitive function performed by consciousness (e.g., cognitive control, pursuit of goals, information broadcasting, reasoning) [11]. Hassin’s conclusions are in line with other studies and related interpretations [12, 13], within a new scientific approach that has been developed, called “the new unconscious” [14].

Furthermore, on the basis of extensive empirical evidence, Hassin outlines that this large amount of unaware operations that the brain is able to perform is not disconnected from but has an important impact on the aware operations. For instance, several findings show that subliminal information can drive executive functions [15] and that subliminal priming of stimuli changes how we feel about them when we are actually aware of them [16]. Other examples confirm the fact that the unconscious plays a significant role in shaping our conscious experiences, like attributions of agency [17], actual choice [18], and even political attitudes [19].

Thus we can legitimately conclude that our aware conscious experience is delayed and reconstructed: it depends on preceding processes taking place without awareness. The brain’s relation with the external environment is not limited to conscious awareness (if that were the case, we could not survive, since our reactions would be far too slow and delayed), and is even in part independent of it.

Moreover, stimuli coming from the external can be evaluated (i.e., assessed in relation to organic developmental needs) at both conscious and unconscious levels. In particular, the unaware evaluation of the stimuli is not free of emotional dimensions: the affective value of the information is largely processed outside our awareness [9], so that the unconscious is not emotionally-free or emotionally-neutral. Even if not universally accepted [20], the concept of unconscious emotion has gained an increasing scientific legitimacy, both in the sense of unawareness of the stimuli eliciting the emotion and of unawareness of the emotion itself [21–25].

In short, the modern sciences of mind, including cognitive science, neuroscience, psychology and philosophy, increasingly reveal the complexity of the unconscious, suggesting that even the deep neuronal distinction between conscious and unconscious levels may need to be revised: stimuli processed without awareness can activate high-level cortical regions [9] (albeit without leading to the functional connectivity with the global neuronal workspace), while aware consciousness might be difficult to segregate from them [26].

## Results

### Ethics of disorders of consciousness

In the ethical discussions about DOCs, two abilities are usually considered as central: experiencing well-being and having interests. In the dominant literature on the ethical dimensions of patients with DOCs these abilities are typically analysed in relation to the residual awareness retained [5, 27–34].

### Well-being

Well-being can broadly be understood as the positive effect related to what makes life good (according to specific standards) for the individual in question. This broad, abstract understanding of well-being need not be assigned only to specific levels or modes of consciousness: for instance, we can meaningfully attribute a so defined well-being also to non-human animals. The only condition for well-being broadly considered is the ability to experience its ‘positiveness’.

Experiencing positiveness is basically an emotional process. As mentioned before, cognitive science provides increasing evidence of unconscious emotions. Particularly, there are some basic emotions (e.g., anger, fear, happiness, disgust) that have a long phylogenetic root and are likely to occur also at a completely unaware level [22]. In other words, the negative or positive reactions to stimuli (i.e., affective reactions) can occur at the unaware level. As some empirical tests show, subjects exposed to subliminal emotional stimuli can be unaware of both the stimuli and the associated positive or negative emotions, even though their behaviour is influenced by them [21].

If the only condition for well-being broadly considered is the ability to experience positive emotions, and these emotions can be experienced at the unaware level, then it is plausible to talk about an unconscious well-being.

The situation becomes more challenging if we try to identify what specifically constitutes the general condition of well-being: depending on the identified constituents, well-being can be related to specific levels or modes of consciousness (e.g., relegated to humans excluding other animals).

Noting that different theories of well-being (both regarding its definition and nature), e.g. hedonism, desire-theories and objectivist theories may likely have diverging views on this, that discussion is beyond the scope of the present paper since well-being broadly defined is sufficient to be ethically relevant.

### Interests

Having an interest in a specific domain can be understood as having a stake in something that can potentially affect what makes our life good within that domain. An interest is what directly and immediately improves life from a certain point of view or within a particular domain, or greatly increases the likelihood of life improvement enabling the subject to realize some good [28]. Even if there is no general consensus on the definition of a good, for the sake of the present discussion we can understand it as what is appropriate for fulfilling a particular need, which of course can be of different kinds. Thus a good is something we can benefit from.

What are the minimal capacities that an individual should have for life to be a good for her/him? Specifically analysing DOCs, Hawkins identifies two possible answers [28]: 1. A life is good if the subject is able to value, or more basically if the subject is able to care. Importantly, Hawkins stresses that caring has no need for cognitive commitment, i.e. for high-level cognitive activities: it requires being able to distinguish something, track it for a while, recognize it over time, and have certain emotional dispositions *vis-à-vis* something. 2. A life is good if the subject has the capacity for relationship with others, i.e. for meaningfully interacting with other people. In her ethical discussion about the best interests of patients with DOCs, Hawkins analyses these two capacities only in relation to awareness without taking into account the possibility that unawareness might also play a role in the adequate understanding of those capacities.

As said above, the brain can be described as evaluative also in its unconscious operations, in the sense that it is able to distinguish relevant inputs, to track and recognize them, and to emotionally react to them in a relevant way. Sensitivity to reward signals is a fundamental element in the learning process, both consciously and unconsciously [35]. Moreover, the unconscious brain is able to interact with its surroundings in a meaningful way and to produce meaningful information processing of stimuli coming from the external environment, including other people [36].

This suggests that unawareness may (at least partly) fulfil both conditions identified by Hawkins for life to be a good for a subject, thus making the unconscious ethically relevant. It is, of course, a different kind of good than what a paradigm cognitive subject with a healthy brain can experience: while we affirm the ethical relevance of unawareness, we leave open the question of its potential ethical value (for example: is the good that an unaware patient with DOC can get from life sufficient for ethically requiring that the patient be kept alive?). We consider this kind of practical and clinical questions important, yet the present paper is not directly aimed at answering them, but rather at suggesting a possible framework allowing a more comprehensive reflection about them.

Focusing specifically on phenomenal consciousness and its relevance for DOCs, Kahane and Savulescu outline that the value of phenomenal consciousness is grounded in the moral significance of interests, which, analogously to well-being, can include hedonic, desiderative and objective elements [30]. Hedonic interests relate to states of suffering or enjoyment; desiderative interests relate to desires´ satisfaction; objective interests relate to having a meaningful existence by possessing relevant goods (e.g., relationship, achievement, knowledge). The authors identify having aware phenomenal consciousness as a necessary condition for all three kinds of interests, because without an aware subjectivity there is no point of view to which such interests can be ascribed [30, 31]. According to this view aware phenomenal consciousness makes a moral difference through its tie to interests, which in turn always require awareness (actual or at some point in time, since at least some interests can extend beyond the extinction of consciousness, as they can extend beyond the life of the beholder [37] ). This definition of interests as necessarily related to awareness is at the root of the “best interests argument” generally employed in ethical discussions about clinical treatment of patients unable to express their preferences, like patients with DOCs [38].

We agree that subjectivity is needed for ascribing interest, but on the basis of recent findings about the unconscious we suggest that awareness is not necessary even if it is sufficient. Rather, in the light of those findings, unawareness is necessary and also sufficient for subjectivity. If, as suggested by Hassin [11], the ability to act unconsciously is something that is developed by practice (i.e., the unconscious is partly shaped by consciousness), then we can reasonably conclude that not only awareness but also unawareness is something highly subjective, in the sense that it is developed in a peculiar and subjective way. We are not all unaware in the same way: there is a “what it is like” to be an unaware subject (e.g., my unconscious could be more emotionally prone to a particular kind of music than yours, so that our brains will react differently to the same music we listen to). Now, if it is reasonable to say that we may have interests also at the unaware level, these interests should be included in the ethical discussion, particularly about patients with DOCs.

Levy says that when approaching patients with DOCs in addition to asking ´is the patient conscious?´ we need to ask ´how is the patient conscious?´ [32]. We agree with this, but disagree with the tendency to place the questions within the limits of awareness ignoring the complexity of unawareness and its ethical relevance.

It is important to note that the existence and ethical relevance of interests at the level of unawareness does not imply that all life is worth living. The kind of good a wholly unaware patient is able to enjoy is not necessarily sufficient for her/his life to be worth living. The determination of when life is worth living depends on numerous other ethical and existential considerations that may vary greatly between individuals, and on other, notably social-legal factors that are beyond our scope here to consider.

## Discussion

We suggest that the ethical relevance of the unconscious can legitimately be inferred from the evidence summarized above about the considerable abilities qualifying the unconscious, which some do not hesitate to describe as comparable to conscious abilities. Two arguments have been provided to support this inference. The first was described as strong because it is based on the assumption that unconscious abilities are comparable to conscious abilities:

*If consciousness is ethically relevant because of what it can enable, and the unconscious may enable comparable things, then the unconscious is also ethically relevant*.

Judging by the evidence summarized above, this is plausible, but remains a debated issue in scientific literature [39–41]. For instance, there is lively debate about the possibility of shifting from subliminal (i.e., unconscious) perception to unconscious cognition (i.e., unawareness of processes and their effects). While Hassin considers unconscious perception to be a subset of the more general and complex phenomenon of unconscious cognition [11], Hesselmann and Moors find this position problematic [40].

A weaker (less controversial) argument is the following:

*If consciousness is ethically relevant, and the unconscious is (at least in part) the result of consciousness, then the unconscious is also ethically relevant*. Or conversely: *if consciousness is ethically relevant, and the unconscious plays an important role in shaping consciousness, then the unconscious is also ethically relevant.*

In other words: the mutual influence of the conscious and unconscious realms yields ethical relevance to them both. In this argument, the focus is not primarily on how similar consciousness and the unconscious are, but rather on their mutual relationship, which is increasingly becoming an object of scientific studies.

### Implications of the ethical relevance of the unconscious for clinical practice

When research on consciousness and the unconscious deepen our understanding, this may have important clinical implications [42, 43]. Enlarging the focus of the ethical analysis of DOCs to include unconscious states has potential clinical implications, to which we would like to draw attention. Acknowledging the ethical relevance of the unconscious may, notably, call for further development and refinement of:

1. diagnostics (including also the need for nosological revision)
2. assessments and interpretations of subjective states (trying to identify unconscious, e.g. emotional, states)
3. adaptations of living conditions (taking into account the possibility of unconscious positive and negative conditions)
4. therapeutic interventions.

If the unconscious is ethically relevant, then diagnostic strategies for the assessment of residual unconscious activities should arguably be implemented. Moreover, the interpretation of subjective states, as well as the adaptation of living conditions, should acknowledge that negative and positive emotions are not necessarily aware. To illustrate, new stimulations of patients’ unconscious perception might be implemented in order to increase their unconscious well-being (e.g., through tactile, olfactory or acoustic inputs), possibly involving those who are close to the patient. Finally, therapeutic interventions on patients with DOCs would be incomplete if they do not involve their unaware level.

End-of-life clinical decisions could be affected by including the unconscious in the ethical assessment. The end-of-life case clearly illustrates that the ethical relevance of the unconscious per se is neutral with regard to specific clinical decisions. One person could consider it right to withdraw life-sustaining care because of the high risk of conscious and/or unconscious negative emotions, while another would consider such withdrawal unjust because of the possibility of residual positive conscious and/or unconscious emotions.

This list of clinical situations where the ethical relevance of the unconscious might have an impact is by no means exhaustive. The point we are making is that acknowledging the ethical relevance of the unconscious makes the task of gaining appropriate clinical assessment and treatment of patients with DOCs even more complex and challenging. We are aware that ideal conditions are not always attainable: there are priorities in healthcare and clinicians need to draw lines as clearly as possible to settle issues arising from their practice. Nevertheless, this should not prevent questions from being raised: reality’s limitations do not constrain theoretical investigations. Even if the actual standards of clinical care of patients with DOCs are the highest possible today, we should be aware that more could be done. And only by raising questions and doubts can we identify the goals to achieve in a close or more distant future.

## Conclusion

The wedge between consciousness and the unconscious, awareness and unawareness, with the resulting clinical and ethical primacy usually attributed to the former does not appear to be in line with contemporary science that reveals the complexity of our unaware mental life and how much it affects our awareness. In light of this knowledge, the exclusive functional and ethical primacy of awareness over unawareness can only be justified by a biased perspective. For this reason, we recommend assessing both aware and unaware abilities in patients with DOCs, since both are ethically relevant. For instance, it could be that the unconscious manifests a particular attitude to enjoy a specific input (e.g., tactile or auditory), and that this should be considered in order to improve the positive emotions the subjects may retain even at the unaware level while reducing the unpleasant ones.

A focus on these aspects should trigger a major change in the ethical discussion about DOCs, which has hitherto been limited to an analysis of residual awareness and its ethical significance. Within this traditional approach, different views have been elaborated concerning the necessary and sufficient conditions for patients with DOCs to be considered conscious in a morally relevant sense, distinguishing different aspects of consciousness and their respective ethical value. Accordingly, the conditions considered critical for the ethical evaluation of patients with DOCs (e.g., well-being and interests) are qualified and assessed only in reference to such residual awareness only. In light of the above discussion, we contest this approach as limited.

It is true that our analysis does not result in concrete suggestions for clinical practice but we consider it important to acknowledge the limitation of the actual default approach to patients with DOCs, stressing the need for a more comprehensive picture of residual mental activity.

## Footnotes

1 In what follows, the unconscious and unawareness, as well as consciousness and awareness are used as synonyms.
